# 

**Published:** 1920-11-13

**Authors:** 


### Matrons' and Sisters' Appointments.

UNION INFIRMARY, WOOLOSTON HOUSE, STOW HILL, NEWPORT, MON.—Miss Minnie Ethel Burrage has been appointed home sister. She was trained at Hackney Union Infirmary, Homerton, and has since been staff nurse under the Territorial Force Nursing Service, and has done private nursing.

HACKNEY UNION INFIRMARY, HOMERTON.—Miss Hetta Forrest has been appointed night superintendent nurse. She was trained at East Dulwich Infirmary, where she was later staff nurse, ward and theatre sister, and night superintendent. She has since done private and military nursing, and has been sister and theatre sister at Graylingwell Hospital, and has held other appointments. Miss Forrest holds the certificate of the Central Midwives Board.

LYNWOOD HOSTEL FOR DISABLED MEN, NEWCASTLE-ON-TYNE.—Miss Mary Dixon has been appointed sister. She was trained at Mile End Infirmary, where she was later staff nurse. She has also served under the British Red Cross Society.

ROYAL SUSSEX COUNTY HOSPITAL, BRIGHTON.—Miss Constance L. Keys-Wells has been appointed sister-housekeeper. She was trained at Charing Cross Hospital and the Royal Hospital for Sick Children, Edinburgh, and has been ward sister and later night superintendent at Charing Cross Hospital; between these two appointments she was sister-in-charge of the Clopton War Hospital, Stratford-on-Avon.

MATERNITY HOME, LANCASTER.—Miss Gertrude Rippon has been appointed matron. She was trained at Camberwell Infirmary, and was subsequently matron of the County Training Home of the Herefordshire County Nursing Association.

PENMORE HOSPITAL, CHESTERFIELD.—Miss E. Martin has been appointed sister. She was trained at the Royal Hospital, Sheffield, and at Leicester Isolation Hospital. She has since been sister at Birmingham City Sanatorium.

BRITISH HOME AND HOSPITAL FOR INCURABLES, STREATHAM.—Miss A. J. Martin has been appointed night sister and Miss C. A. Palmer sister. Miss Martin was trained at Liverpool Royal Infirmary, and has since been sister-in-charge of the Turner Memorial Home, Liverpool, and has worked under the Joint War Committee for four years. She is a member of the College of Nursing. Miss Palmer was trained at Birmingham Infirmary, Dudley Road, and has since been ward sister at Bethnal Green Infirmary, a Queen's nurse, theatre sister at Khartoum Government Hospital, and assistant county superintendent under the East Sussex County Nursing Federation.

ESSEX COUNTY HOSPITAL, COLCHESTER.—Miss H. Williams has been appointed ward sister. She was trained at the Guest Hospital, Dudley, and has since been staff nurse at the Hospital for Women, Soho Square, and sister under the Territorial Force Nursing Service. She holds the certificate of the Central Midwives Board.

